# [Corrigendum] Circular RNA 0001666 inhibits colorectal cancer cell proliferation, invasion and stemness by inactivating the Wnt/β‑catenin signaling pathway and targeting microRNA‑1229

**DOI:** 10.3892/ol.2025.14885

**Published:** 2025-01-13

**Authors:** Fei Bai, Chaohui Zuo, Yongzhong Ouyang, Ke Xiao, Zhuo He, Zhi Yang

Oncol Lett 23: 153, 2022; DOI: 10.3892/ol.2022.13273

Subsequently to the publication of the above article, the authors contacted the Editorial Office to explain that certain of the Transwell cell invasion assay shown in Fig. 2D on p. 5 had been assembled in this figure incorrectly (the data in question were subsequently included in an article featuring some of the same authors that was submitted to the journal *Frontiers in Oncology*). However, the authors had retained their original data, and were able to identify how these errors occurred.

The revised version of Fig. 2, showing data for Fig. 2D from one of the repeated experiments, is shown on the next page. Note that the errors made in compiling this figure did not affect either the results or the conclusions reported in this paper, and all the authors agree to the publication of this Corrigendum. The authors thank the Editor of *Oncology Letters* for allowing them the opportunity to publish this Corrigendum, and apologize both to the Editor and to the readership of the Journal for any inconvenience caused.

## Figures and Tables

**Figure 2.**
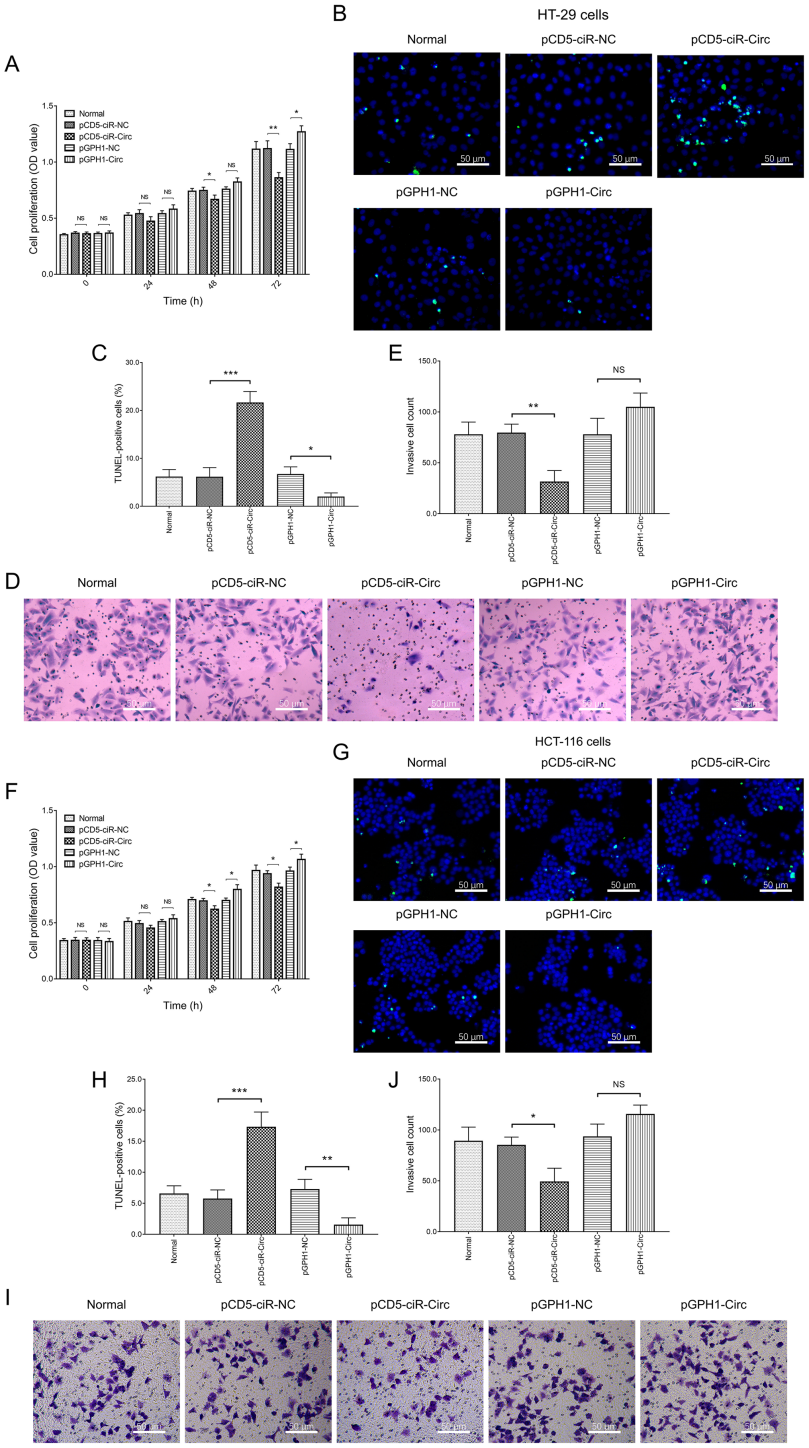
Cell proliferation, apoptosis and invasion following circ_0001666 overexpression or knockdown. (A) Proliferation in the HT-29 cells following transfection. (B and C) Cell apoptosis rate and (D and E) the number of invasive cells in the HT-29 cell line following transfection. (F) Proliferation in the HCT-116 cell line following transfection. (G and H) Apoptosis rate and (I and J) the number of invasive cells in the HCT-116 cell line following transfection. *P<0.05, **P<0.01, ***P<0.001. NS, not significant; circ, circular RNA; NC, negative control. pCD5-ciR-Circ represented circ_0001666 overexpression plasmids, pCD5-ciR-NC represented overexpression NC plasmids. pGPH1-Circ represented circ_0001666 knockdown plasmids, pGPH1-NC represented knockdown NC plasmids.

